# Improved annotation of protein-coding genes boundaries in metazoan mitochondrial genomes

**DOI:** 10.1093/nar/gkz833

**Published:** 2019-10-04

**Authors:** Alexander Donath, Frank Jühling, Marwa Al-Arab, Stephan H Bernhart, Franziska Reinhardt, Peter F Stadler, Martin Middendorf, Matthias Bernt

**Affiliations:** 1 Center for Molecular Biodiversity Research (ZMB), Zoological Research Museum Alexander Koenig (ZFMK), Adenauerallee 160, D-53113 Bonn, Germany; 2 Inserm, U1110, Institut de Recherche sur les Maladies Virales et Hépatiques, 3 Rue Koeberlé, F-67000 Strasbourg, France; 3 Université de Strasbourg, 4 Rue Blaise Pascal, F-67081 Strasbourg, France; 4 Bioinformatics, Department of Computer Science, Universität Leipzig, Härtelstraße 16-18, D-04107 Leipzig, Germany; 5 Doctoral School of Science and Technology, AZM Center for Biotechnology Research, Lebanese University, Tripoli, Lebanon; 6 Interdisciplinary Center for Bioinformatics, University of Leipzig, Härtelstraße 16-18, D-04107 Leipzig, Germany; 7 Competence Center for Scalable Data Services and Solutions Dresden/Leipzig, German Centre for Integrative Biodiversity Research (iDiv), and Leipzig Research Center for Civilization Diseases, Universität Leipzig, Leipzig, Germany; 8 Max Planck Institute for Mathematics in the Sciences, Inselstraße 22, D-04103 Leipzig, Germany; 9 Fraunhofer Institut for Cell Therapy and Immunology, Perlickstraße 1, D-04103 Leipzig, Germany; 10 Department of Theoretical Chemistry, University of Vienna, Währingerstraße 17, A-1090 Wien, Austria; 11 Santa Fe Institute, 1399 Hyde Park Rd., Santa Fe, NM 87501, USA; 12 Swarm Intelligence and Complex Systems, Department of Computer Science, Universität Leipzig, Augustusplatz 10, D-04109 Leipzig, Germany; 13 Helmholtz Centre for Environmental Research – UFZ, Young Investigators Group Bioinformatics and Transcriptomics Permoserstraße 15, D-04318 Leipzig, Germany

## Abstract

With the rapid increase of sequenced metazoan mitochondrial genomes, a detailed manual annotation is becoming more and more infeasible. While it is easy to identify the approximate location of protein-coding genes within mitogenomes, the peculiar processing of mitochondrial transcripts, however, makes the determination of precise gene boundaries a surprisingly difficult problem. We have analyzed the properties of annotated start and stop codon positions in detail, and use the inferred patterns to devise a new method for predicting gene boundaries in *de novo* annotations. Our method benefits from empirically observed prevalances of start/stop codons and gene lengths, and considers the dependence of these features on variations of genetic codes. Albeit not being perfect, our new approach yields a drastic improvement in the accuracy of gene boundaries and upgrades the mitochondrial genome annotation server MITOS to an even more sophisticated tool for fully automatic annotation of metazoan mitochondrial genomes.

## INTRODUCTION

The precise and consistent annotation of genes is a prerequisite for all downstream analyses and in particular for comparative genomics and phylogenetics. This seems to be an almost trivial task taking into account the small number of protein-coding genes (PCGs) encoded in a mitochondrial genome (mitogenome). It is indeed not difficult to determine the identity and *approximate* position of these genes. Due to peculiarities of mitochondrial genetics it remains a challenging problem, however, to determine the precise start and end positions. Given the rapid increase in the number of sequenced mitogenomes—the NCBI organelle genome database lists >8000 animal species at the time of writing (September 2018)—a careful manual annotation of mitogenomes has become infeasible. This requires precise methods that automate this task and reduce the burden of manual curation. Here, we describe a highly accurate and fully automated procedure for annotating the boundaries of PCGs in metazoan mitogenomes.

Mitochondrial genes are expressed from polycistronic transcripts. For example, the number of transcriptional units that have been described is three for vertebrates (1) and five for arthropods (2). According to the tRNA punctuation model (3) tRNA elements serve as excision points to generate mRNAs of the PCGs encoded by these units. Multiple genetic codes have been described for mitogenomes. The variations of the genetic code affect the start and stop codons in particular, see e.g. (4). In mitogenomes, many PCGs have been described to feature only incomplete stop codons (such as TA or T) (3) for which complete stop codons are generated by polyadenylation, see e.g., (5). Furthermore, in some invertebrate taxa it has been reported that PCGs often start with *non-canonical* start codons, i.e. codons that are not defined as start codon in the genetic code tables. But for a few cases alternative possibilities to non-canonical start codons have been described. The initiation codon of the cytochrome c oxidase subunit I (*cox1*) gene in *Drosophila*, for instance, is out of frame and requires a post-processing of the quadruplet AUAA by frameshift or editing (6), see (7) for a review. Even if non-canonical start codons are mainly described for invertebrates, the frameshift mechanism recently gained support for the human mitochondrial genome (8).

RefSeq and GenBank (9) are the major source of annotated mitochondrial genome sequences. Problems with consistency and quality of annotations in these databases have been described repeatedly (10,11) and have been approached in two different ways.

The first focuses on the manual improvement and reannotation of existing database entries. The OGRe database (12) incorporates corrections that have been described in the literature. The manual reannotation performed in MitoZOA (13) follows defined rules for detection and correction of errors in databases. The major problem with such manual curation approaches is that they seem to be unsustainable because they do not scale with the ever faster growing amount of available mitochondrial genomes.

A second group of methods implement *de novo* annotation approaches. The first published computational method to annotate mitogenomes was DOGMA (14). It uses BLAST (15) for detecting PCGs as well as ribosomal RNAs (rRNAs) and applies tRNAscan-SE (16) for annotating tRNAs. Potential ends of genes are selected by the user. A fully automated method for the *de novo* annotation of mitogenomes has been introduced for the first time with MITOS (11). In contrast to DOGMA, MITOS uses curated covariance models to detect non-coding RNAs. For tRNAs, it uses the MiTFi approach (17). PCGs are identified by applying a BLAST-based strategy with a subsequent identification of exact gene boundary positions in the proximity of the approximate positions that are estimated from the results of this strategy. With MitoAnnotator (18) an automated approach for the annotation of fish mitogenomes has been described that has recently been extended by methods for metabarcoding analysis (19). It also employs MiTFi for detecting tRNAs but uses BLAST for both, PCGs and rRNAs. Boundaries of PCGs are determined by a set of manually defined rules that are tailored to fish mitogenomes.

Here, we present a novel probabilistic method to predict the positions of start and stop codons of mitochondrial PCGs. It takes into account codon frequencies and length distributions of PCGs in reference annotations and distance estimations to corresponding gene boundaries. To this end, we performed a comprehensive analysis of the codons in the RefSeq annotations of metazoan mitogenomes. To test the performance of our novel method, we compared its results and the results of the method originally implemented in MITOS to the mitogenome annotations in NCBI RefSeq, a set of manually curated annotations (MitoZOA), and the results of a *de novo* annotation by MitoAnnotator.

## MATERIALS AND METHODS

### Computation of approximate gene positions

Initial predictions of the PCGs are computed using the hidden Markov models (HMMs) and methods from (20), which in turn make use of HMMER (21). These models have been generated based on the amino-acid sequences of the PCGs in RefSeq 63 with an automated method that takes their phylogenetic classification into account. It was shown that the predictions made with these models are specific and sensitive, but lack a precise annotation of the start and stop codon positions (20). Therefore, we subsequently improve the start and stop codon positions of these initial annotations. We first briefly describe how the gene boundaries were selected in the original implementation of MITOS and then introduce our newly developed approach in detail.

### Prediction of start and stop codon positions in MITOS

The original implementation of MITOS employs a very simple method to predict start and stop codons of PCGs. Given approximate start and stop positions (provided, e.g. by a BLAST search) the proximity (per default in a range of ±6 amino acids) is checked for start and stop codons, respectively, using the genetic code tables of the NCBI Taxonomy (22) (https://www.ncbi.nlm.nih.gov/Taxonomy/Utils/wprintgc.cgi). If no valid start or stop codon can be identified, MITOS chooses the corresponding approximate position as gene boundary.

### Improved probabilistic prediction of start and stop codon positions

Given an approximate gene position, all codons between the adjacent upstream stop codon and the (inframe) center point of the initial prediction are considered as potential start sites. Analogously, codons between the (inframe) center and the nearest downstream stop codon are taken into account as potential stop codons positions. For the determination of these search ranges, full stop codons according to the NCBI genetic code tables are considered. In the following, we denote by *S* and *E* the sets of positions that are evaluated as potential start and stop positions, respectively.

The most probable start and stop positions of a gene are determined by maximizing the product of three factors over all possible candidate positions in *S* and *E*, respectively: (1) a factor (δ) that depends on the distances of the candidate positions to the estimated start or stop position inferred by comparison with the query model, (2) the (empirical) probability that the codon at the candidate position is a start or stop codon (ϕ), (3) and the (empirical) probability of the resulting gene length (λ). These factors are quantified as follows:

To determine δ, the start (*s*_*s*_) and stop positions (*s*_*e*_, where *s*_*e*_ > *s*_*s*_) in the target sequence and the start and stop position in the query HMM (*q*_*s*_ and *q*_*e*_, respectively) are used as described in (20), see also Supplementary Text 1.1 and Supplementary Figure S1 for a more detailed explanation. For a given sequence position *p* in the interval *s*_*s*_ ≤ *p* ≤ *s*_*e*_ the *relative start position r*_*s*_(*p*) and the *relative stop positionr*_*e*_(*p*) are computed as

$$\begin{eqnarray*} r_s(p) &=& q_s + \frac{q_e-q_s}{s_e - s_s} (p - s_s)\nonumber \\ r_e(p) &=& (l_q - q_s) + \frac{q_e-q_s}{s_e-s_s} ( p - s_s ), \end{eqnarray*}$$

where *l*_*q*_ is the length of the query HMM. The values for δ are then computed separately for the start and stop position as

$$\begin{eqnarray*} \delta _s(p)=1-\frac{r_s(p)}{l_q} & \ \ \text{and} \ \ \delta _e(p)=1-\frac{r_e(p)}{l_q}. \end{eqnarray*}$$

That is, the closer the value of δ is to 1, the closer is the position to the gene boundary. Since the predicted PCGs of the methods from (20) are typically too short, we set δ_*s*_(*p*) = 1 and δ_*e*_(*p*) = 1, respectively, for positions $p\not\in [s_s,s_e]$.

The contribution of the length distribution is computed as an empirical (two-sided) *p*-value λ(*l*) to observe a gene with length *l*. More precisely

$$\begin{eqnarray*} \lambda (l) = 2 \times \frac{\min (L_{\le ,l}, L_{\ge ,l} )}{ L_{\le ,l} + L_{\ge ,l}}, \nonumber \end{eqnarray*}$$

where *L*_≤, *l*_ and *L*_≥, *l*_ are the number of species in RefSeq 63 that have the same genetic code and where the gene in question has a length of at least (or at most, respectively) *l*.

The probability ϕ_*s*_(*p*) of the codon at position *p* to be a start codon is estimated from the frequency of how often the codon is used as a start codon in the RefSeq 63 annotations. To account for possible annotation errors, start codons that appear with a frequency <0.01 are not considered. The probability ϕ_*e*_(*p*) of a codon to be a stop codon is calculated accordingly. However, all codons that are annotated as inner codon with a frequency of at least 0.001 are ignored here. The probability ϕ_*e*_(*p*) is also computed for incomplete stop codons (T and TA). Compared to an unambiguously determined full stop codon, incomplete stop codons are more likely found by chance (four and 16 times, respectively). Therefore, the frequency is adjusted as follows: If only one nucleotide is missing (incomplete stop codon TA), ϕ_*e*_(*p*) is divided by 3 because TAA is already considered. If two nucleotides are missing (incomplete stop codon T), ϕ_*e*_(*p*) is divided by 12 because TAN is already accounted for in the calculation of ϕ_*e*_(*p*) for complete stop codons.

The final gene boundaries are now computed as the pair (*s, e*), where *s* ∈ *S* and *e* ∈ *E*, that maximizes the product of δ, ϕ, and λ, formally,

$$\begin{eqnarray*} \mathop {\rm argmax}_{s\in S, e\in E}\ \delta _s(s)\cdot \phi _s(s)\cdot \delta _e(e)\cdot \phi _e(e)\cdot \lambda (e-s+1). \end{eqnarray*}$$

The values of ϕ and λ are determined for each corresponding gene and genetic code. This is necessary because codon frequencies and gene lengths vary substantially between the taxa that share the same genetic code (see Supplementary Figure S6). Ignoring these variations could lead to considerable under- or overestimations of species-specific gene lengths during annotation.

In order to reduce the number of pairs (*s, e*) that need to be evaluated, only positions *p* in *S* (and *E*, respectively) are considered for which ϕ_*s*_(*p*) > 0 (and ϕ_*e*_(*p*) > 0, respectively). To further reduce the search space, combinations of start and stop codon positions that imply the inclusion of a tRNA-gene are forbidden. To this end, the set of tRNA-genes consisting of the best prediction for each type as determined with MiTFi (17) is considered.

Our novel gene prediction method for gene boundaries is able to identify canonical start/stop codons, non-canonical start codons, and incomplete stop codons if they lie within the analyzed range of positions. In the rare cases where no suitable (incomplete) codons can be found, the initial boundaries as predicted by HMMER are selected.

### Performance evaluation

For the performance evaluation of the new method we computed annotations for all complete mitochondrial genomes in RefSeq 89 which were not present in RefSeq 63 and compared them with the reference annotations provided by RefSeq 89 and MitoAnnotator (downloaded from http://mitofish.aori.u-tokyo.ac.jp on 1 September 2018). The annotations computed by MitoAnnotator refer to sequences that are circularly shifted to begin with the *trnF* locus. In addition, we compared our annotations with the annotations of MitoZOA version 10 (13). MitoZOA, however, contains only 9 sequences that are contained in RefSeq 89 which were not present in RefSeq 63. Thus, we reannotated the RefSeq 63 entries and compared only those results with the annotations in MitoZOA. The number of mitogenomes in the four reference data sets is given in Table 1.

**Table 1.** Number of complete mitogenomes in the different data sets analyzed here

Data set	No. of mitogenomes
RefSeq 63	3842
RefSeq 89*	4264
MitoZOA^+^	2482
MitoAnnotator*	2618

*Only mitogenomes present in RefSeq 89 but not in RefSeq 63. ^+^Only mitogenomes that are also present in RefSeq 63.

We compared start and stop codon positions as provided by the CDS features in the reference annotations with the annotations computed by the new method presented here. For this only true positive predictions as defined by (20) were considered (see Supplementary Table S3 for an evaluation of the precision of the annotation). In short, pairs of PCGs computed by our new method and a reference annotation are determined that have the bidirectional largest overlap. Note that the HMM-based method of (20) has been shown to be very precise, i.e. it results in only very few false positives and false negatives. For the calculation of the true positives, the differences of the start positions computed by our new method and the reference annotation of the gene are determined such that positive values correspond to cases where the predicted position is outside of the gene in the reference annotation. Negative values correspond to cases where the predicted position is within the gene boundaries of the reference annotation.

## RESULTS

In the following, we analyze the codon frequencies and gene lengths of mitochondrial PCGs that can be inferred from the annotations of the PCGs in RefSeq 63. After that, we present an evaluation of the precision of our new method to predict start and stop positions.

### Codon frequencies in mitochondrial PCGs

The NCBI Taxonomy provides a comprehensive listing of the different genetic codes. Currently, it contains seven code tables that have been described for metazoan mitochondrial genomes. The number of the available mitogenomes, however, is unevenly distributed over the different mitochondrial code tables (analogous to the biased taxonomic distribution). While code tables 2 (vertebrate mitochondria) and 5 (invertebrate) each apply to at least 25% of the RefSeq mitogenomes, each of the other code tables is used in <4% of the RefSeq mitogenomes. Most notably, the ascidian code table 13 is used in only 20 mitogenomes and the pterobranch code table 24 applies only to a single mitogenome. The alternative flatworm mitochondrial code table 14 is not used in RefSeq 63 (see Supplementary Table S2 for details).

**Table 2.** Statistics of the precision of the gene boundary predictions for the method originally implemented in MITOS (old) and the new method presented here (new) for the reannotation of mitogenomes present in RefSeq 89 but not in RefSeq 63

	RefSeq 89 (old)		RefSeq 89 (new)	
	Start	Stop	Start	Stop
μ	8.61	5.93	4.35	2.04
σ	59.18	50.29	48.84	41.00
*d* = 0	58.99%		74.35%	
*d* ≤ 3	69.81%		86.43%	
*d* ≤ 9	75.52%		90.14%	
*d* ≤ 30	89.35%		95.75%	

Top part: mean (μ) and standard deviation (σ) of the absolute values of the differences for start and stop position (in base pairs (bp)). Bottom part: percentage of the PCGs where the maximum difference (*d*) between annotated and predicted start and stop positions is less than or equal to 0, 3, 9 and 30 bp, respectively.

#### Start codons

There are several codons or codon boxes (i.e. sets of codons that differ only in their last position and are treated equivalently in translation) that have, according to the NCBI code tables, the potential to initiate translation in mitochondria:

1. While all codons in the 4-fold degenerate codon box ATN can initiate translation according to code tables 2, 4 and 5, this is only partially the case in code tables 9 (ATG), 13 (ATR), and 24 (ATK).
2. TTG is present as a start codon in all mitochondrial code tables with the exceptions of code tables 2 and 9. In code table 4 TTG is extended to TTR.
3. The start codon GTG is used in all but code table 14.
4. Code tables 4 and 24 define CTG as additional start codon.

**Figure 1.**
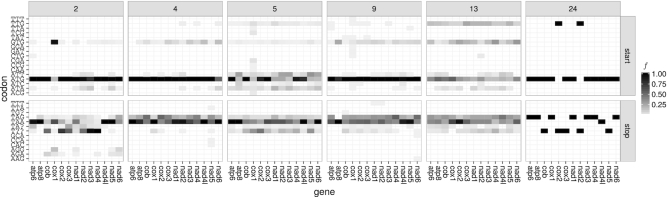
Overview of the annotated start codon (upper panel) and stop codon (lower panel) frequencies (*f*) ≥ 0.01 per gene and genetic code table inferred from the annotations in RefSeq 63; full data is given in Supplementary Figure S7.

The frequencies of start codons that are annotated in RefSeq 63 show strong specific differences between PCGs and the sets of mitogenomes that share the same code table (see Figure 1). In general, codon ATG is the most frequently annotated start codon. For invertebrates and ascidians (code tables 5 and 13, respectively), however, the other three codons of the codon box ATN are also frequently annotated; in some PCGs even to a larger extent than ATG, e.g., ATT in invertebrate *nad3* (code table 5), see Figure 1. Interestingly, some of the start codons defined in the NCBI genetic code tables are either virtually absent (frequency (*f*) < 0.01) in the annotations, e.g. TTA and CTG in coelenterates (code table 4) (note that for those mitogenomes also TTG is annotated as start with low frequency in a few PCGs only), or are never annotated, e.g., CTG, ATT, and GTG in Pterobranchia (code table 24).

A notable difference between the definitions in the NCBI genetic code tables and the RefSeq annotations are non-canonical start codons. For echinoderms and flatworms (code table 9) only one and for ascidians (code table 13) only ATR are defined as start (see above). The annotated start codons, however, support the complete codon box ATN as start (*f* ≥ 0.01 in up to eight of the 13 mitochondrial PCGs). Furthermore, TTG is not defined as a start codon in code table 9 but is annotated in five PCGs with *f* ≥ 0.01.

Generally, *cox1* seems to be a hotspot for non-canonical start codons in annotations of invertebrate mitogenomes (code table 5) and to a lesser extent also in echinoderms and flatworms (code table 9). In taxa using code table 5, the annotated non-canonical start codon TTA extends the defined TTG start codon to a TTR start codon box and the non-canonical start codons ACG, CAA, CCG, CGA and TCG can be found in the RefSeq annotations. Whereas the codons TTT, TAT, GGA, GAT and CTG are annotated as non-canonical start codons in echinoderm and flatworm mitogenomes (code table 9). Moreover, CTG is annotated as start codon in vertebrate and ascidian mitogenomes (code tables 2 and 13, respectively) for *atp6* and *nad2*. Note, that this codon is also defined as start codon in code tables 4 and 24. Except for *cox1* (9.8%), *atp6* (1.49%), and *nad4l* (1.27%), the frequency of annotated non-canonical start codons is less than 1%. For *cox1* most cases of annotated non-canonical start codons (312 out of 338) appear within Arthropoda. According to the RefSeq annotations, the use of non-canonical start codons does not seem to be conserved within subphyla or superclasses, i.e. they are annotated only in a subset of the species within Chelicerata (14 out of 73 species), Crustacea (53 out of 118), Hexapoda (240 out of 467), and Myriapoda (5 out of 14). Considering the lowest taxonomic ranks (according to the taxonomy string given in the GenBank files), non-canonical start codons are occasionally not monophyletic: of the genus *Amblyomma* (Arthropoda: Arachnida) one out of five species have non-canonical start codons. Other examples are the genera *Cherax* (Arthropoda: Malacostraca) (4 out of 5 species have non-canonical start codons), *Gomphocerus* (Arthropoda: Insecta) (2 out of 3) and *Locusta* (Arthropoda: Insecta) (3 out of 4).

#### Stop codons

According to the NCBI genetic code tables, TAR are stop codons throughout metazoan mitogenomes (with the exception of the alternative flatworm code table 14 which lists only TAG). Additionally, AGR are defined as stop codons in vertebrate mitogenomes (code table 2). All stop codons that are allowed by these 2-fold degenerate codon boxes are found in the RefSeq annotations. However, similar to start codons, their relative frequency varies substantially between code tables and PCGs. For vertebrates, annotations of the additional stop codons AGR are restricted to a subset of PCGs and appear with lower frequency than TAR stop codons. Several other codons are annotated in RefSeq as stop codons, although with low frequencies – in particular TTT (which was also found as an annotated start codon), GCA and AAG in echinoderms and flatworms (code table 9) and TTA, CAC and CCA for *nad5* in invertebrates (code table 4).

Incomplete stop codons T and TA are annotated in RefSeq throughout Metazoa. For species with code table 4 they are annotated with very low frequencies and for a very small number of PCGs only. Apparently, T is present more often than TA.

#### Internal codons

In the following, we analyze the frequencies of codons between a start and a stop codon of annotated PCGs in RefSeq because in our new method they are used to determine the codons that are accepted as stop codons. That is, codons are accepted as potential stop codons only if they appear as internal codons with very low frequency.

**Figure 2.**
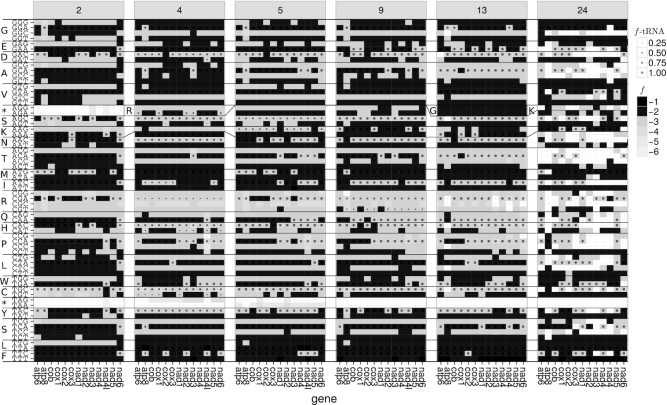
Usage of internal codons (inframe codons between annotated start and stop positions) in different coding tables. Shading of the boxes represents the frequency (*f*) of the internal codons according to PCGs annotated in RefSeq 63 (expressed as ⌊*log*_10_*f*⌋). Frequencies of the corresponding tRNA anticodons (*f*-tRNA) and accepted amino acid are indicated by the circle sizes. Changes of encoded amino acid between code tables are indicated by changes of the line profile or their single-letter code (e.g. AGR codes for stop in code table 2, for arginine in code table 4, and—together with AGY—for serine in code table 5).

The stop codons TAR and AGR are found within PCGs with very low frequency (*f* < 10^−4^) (see Figure 2). In vertebrates (code table 2), the codon AGR appears internally with very low frequency within *nad3* and *nad4l*. The 83 internal TAG codons in coelenterate mitogenomes (code table 4) are found in four out of the six mitogenomes of the genus *Clathrina* (Porifera: Calcarea) (RefSeq accessions NC_021112 – NC_021115) that are included in RefSeq 63 and in a single gene of *Cubaia aphrodite* (Cnidaria: Hydrozoa). The 231 internal TAA codons in invertebrate mitogenomes (code table 5) are found in only a small number of species (one mollusk, four hexapods, one crustacean, and one nematode).

We further analyzed the appearance of TAR stop codons in all 65526 metazoan mitochondrial genomes currently (2017-06-11) contained in GenBank. The codon TAA was found in 521 mitogenomes, of which only 248 use this codon at least 10 times. Only few of these have code table 14 assigned to them (one annelid, one cestode, three nematodes, and one trematode). With 157 occurrences, TAA is the most abundant codon in the mitogenome of *Radopholus similis* (Nematoda: Chromadorea) (NCBI accession number NC_013253 = FN313571) and thus code table 14 has been assigned to this species (23). For many other species only approximate gene positions are annotated in the GenBank files, e.g. ‘<4787..>5822’ for *nad2* in *Hydractinia polyclina* (Cnidaria: Hydrozoa) (LN901196) which has a total of 60 internal TAA codons. Oddly, code table 14 has also been assigned to two different platyhelminths (KT008005 and KX943545). However, TAA appears as an internal codon only five times in KT008005 (and never in KX943545).

TAG is counted as internal codon in 470 mitogenomes and is most frequent (61 copies) in *Physalia physalis* (Cnidaria: Hydrozoa) (KT809328), for which only approximate gene positions are annotated for most of its PCGs.

We observe differences in the codon usage within codon boxes as well as between PCGs and taxa (see Figure 2). The most frequent tRNA anticodons correspond to codons ending with A or C if the codon box does not include a codon ending with A (see Figure 2). The only exception is the codon box for methionine for which the codon matching the present anticodon ends with G. Interestingly, this exception appears for the most frequently used start codon ATG. Furthermore, codons that appear most frequently for a given amino acid typically match the corresponding tRNA anticodon that is coded in the mitogenome.

### Precision of predicted start and stop positions

In the following, we present the performance of the new probabilistic method for the prediction of gene boundaries and the method originally implemented in MITOS by comparing their results to the annotations in RefSeq, MitoZOA and MitoAnnotator . While a comparison with reference data and the results of other automatic prediction methods certainly is a good performance indicator, an evaluation with other independent data is desirable. To this end we consider polyadenylated transcripts from the mitochondrion. Using RNA-seq data for six phylogentically diverse species, we determined the exact position of the polyadenylation, which marks the 3′ end of the transcript. In addition, polyadenylation may complete missing stop codons. The analysis shows only very small deviations of the positions of the polyA sites to the 3′ gene boundaries annotated in RefSeq and those predicted by our method (Supplementary Text 1.3 and Figures S1, S2 and S3). In addition, a detailed case study of the performance of the novel annotation method based on RNASeq data of the mitogenome of the bank vole *Myodes glareolus* (Chordata: Mammalia) (NC_024538 = KF918859) (24) can be found in the Supplementary Text 1.3, Table S1 and Figures S4 and and S5. Whereas the data set is much to small to be conclusive it indicates that both annotation sets have a high precision and also that a comparison with the reference data set is a good quality indicator.

**Figure 3.**
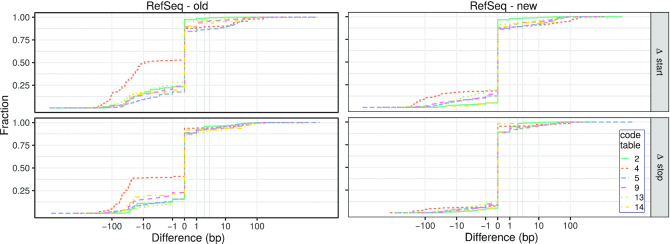
Cumulative plot of the differences (in base pairs) of the start (Δ start) and stop positions (Δ stop) predicted with the method originally implemented in MITOS (left) and the new method presented here (right) with respect to the reference annotations for those entries that are present in RefSeq 89 but not in RefSeq 63. Positive (negative) values correspond to predictions outside (inside) of the annotation. Differences are shown on an inverse hyperbolic sine scale ($f(x)=\mathop {\mathrm{arcsinh}}x)$). For a comparison to RefSeq 63, see Supplementary Figure S8.

Results show that the method originally implemented in MITOS predicts start and stop positions that differ to their RefSeq annotation for more than 40% of the PCGs (see Table 2, Figure 3, Supplementary Figure S8, and Table S4. For some taxa, e.g., those with code table 4, the positions differ even in most cases. This problem remained unnoticed in MITOS because differences to reference annotations were analyzed only jointly for all Metazoa. Since the sample set is dominated by vertebrate mitogenomes, systematic, but comparably rare biases were not identified during the performance evaluation of MITOS.

In most cases, the positions predicted by our new method are in agreement with RefSeq (77.5% and 75.8% of the start and stop positions, respectively). Still, the precision for vertebrates (code table 2) is clearly better compared to the results for non-vertebrates (code tables 4, 5, 9 and 13). Here, our new adaptive method profits from the large fraction of vertebrate mitogenomes in the training set (Supplementary Table S2. Note that a considerable proportion of predicted stop codon positions differ by only one or two base pairs from the reference annotation. This is because our new method annotates the last position of incomplete stop codons (i.e. T for T and A for TA, respectively). This is handled differently in many RefSeq annotations in which complete stop codons are annotated, although they are clearly incomplete. Interestingly, only 80 PCGs (distributed over 61 mitogenomes) are annotated in the 4264 metazoan mitogenomes of RefSeq 89 (excluding the RefSeq 63 data) where the positions of the gene feature and the CDS feature differ. In contrast, our new method annotates 6725 of the 54045 (12.4%) PCGs in the RefSeq 89 data set (excluding the RefSeq 63 data) for which the gene boundaries differ by one or two base pairs from the corresponding reference annotation (compared to 40941 PCGs where the RefSeq annotation and the prediction of our new method match).

#### Comparison with other reference data sets

In addition to a comparison with RefSeq, we compared our predictions to those of MitoZOA and MitoAnnotator. The data in MitoZOA contains manually curated annotations of metazoan mitogenomes. MitoAnnotator promises a precise annotation of fish mitogenomes that is based on BLAST and a manually curated set of rules for the annotation of start and stop codon positions.

The comparison of our new method to the annotations by MitoZOA shows similar results as the comparison to RefSeq: the absolute difference of annotated and predicted start codon positions is on average 6.54 bp (σ = 92.14 bp) and 3.74 bp (σ = 77.09 bp) for stop codon positions (see Supplementary Table S5 and Figure S10). For 63.40% and 80.04% of the predictions by our novel method the absolute differences to the MitoZOA annotations are zero and less than four base pairs, respectively. Note that the improvements of the RefSeq annotations by MitoZOA do not include modifications of the start and stop positions in the case of non-canonical start or incomplete stop codons. Only a note is added if non-canonical start codons are detected.

**Figure 4.**
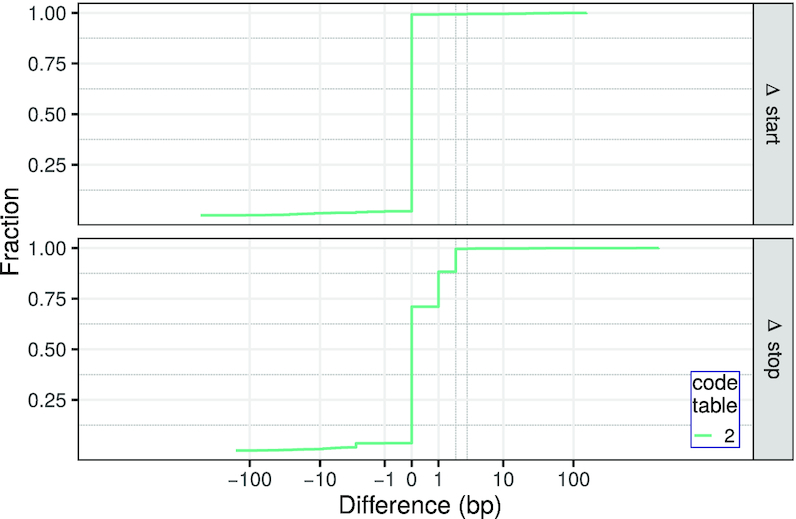
Cumulative plot of the differences (in base pairs) of the predicted start and stop codon positions and the positions annotated in MitoAnnotator for genomes present in RefSeq 89 but not in RefSeq 63.

The comparison with MitoAnnotator is based on 1267 fish mitogenomes that are present in MitoAnnotator and RefSeq 89 but not in RefSeq 63. The predictions of start and stop codon positions by MitoAnnotator and our new method show a considerably higher agreement for both (start: μ = 0.67 bp, σ=9.03 bp; stop: μ = 1.19 bp, σ = 19.91 bp) (see Figure 4, Supplementary Table S5, and Figure S9). The difference of the start and stop positions is zero and less than four base pairs for 65.99% and 96.21% of the PCGs, respectively. This nicely shows that the prediction of start and stop codons of our new method performs as well as employing manually curated rules. Note that MitoAnnotator annotates incomplete stop codons for the CDS features (the length of 16 903 features is a multiple of 3, whereas 11 340 and 5778 have a remainder of 1 and 2, respectively). Thus, our new method shows a considerable number of cases where the annotations differ by one or two base pairs to the MitoAnnotator annotation.

## DISCUSSION

Problems of MITOS to annotate the correct start and stop codon positions have been reported repeatedly (7,18,25). These inaccuracies are mostly caused by the rather unsophisticated strategy in the previous release of MITOS, which limits the search range to six amino acids around the initial prediction, and the strict use of the NCBI genetic code tables. Our empirical survey of annotated start, stop and internal codons shows, however, that the definition of the NCBI genetic code tables and their usage in RefSeq are not always consistent with the available annotations (which due to the size of the available sample of mitogenomes may be considered as a reasonable approximation to the genetic reality). In particular, the tables do not cover non-canonical start and incomplete stop codons. Furthermore, gene- and code table-specific differences in codon usage frequencies are not reflected due to the binary nature of the NCBI genetic code tables.

In order to resolve these shortcomings, we have developed a method that makes use of the empirical start and stop codon frequencies of PCGs annotated in RefSeq in a way that treats the different PCGs and the code tables separately. The frequencies of annotated codons show remarkable differences between organisms that use different variants of the genetic code.

### 

#### Evaluation of annotated start and stop codons

Overall, the most frequently annotated start codon in RefSeq mitogenomes is ATG. Other canonical start codons, e.g., GTG or TTG, are rarely annotated. However, non-canonical start codons are frequently annotated as well, in particular for the gene *cox1* in invertebrate mitogenomes. These are not confined to well-separated monophyletic groups, in particular in the case of arthropods. This observation could be explained by a high level of tolerance for certain non-canonical start codons in arthropods that renders their recurrent emergence essentially neutral. Alternatively, non-canonical start codons could be the result of a common parallel evolution of the translation machinery that are only mis-annotated as (infrequent) non-canonical start codons.

The most frequent metazoan stop codons annotated in RefSeq mitogenomes are either TAG or TAA. The codons AGG and AGA can be found as annotated stop codons in vertebrates, although their use is restricted to specific PCGs. However, incomplete stop codons, i.e., T and TA, that are post-transcriptionally adenylated are frequently observed. At present, these are not annotated consistently in RefSeq. In some cases, a complete codon is annotated even if it is not a stop codon according to the designated translation table. We suggest that it would be good practice to differentiate between gene features—which should not contain polyadenylated positions—and CDS features—which should contain these positions.

Currently, incomplete stop codons are preferred over small overlaps with adjacent downstream genes in RefSeq annotations. On the other hand, RefSeq 63 contains 2085 annotations (in 1342 species, see also Supplementary Figure S11) of genes overlapping more than 10 bp. For instance, *atp8* and *atp6* overlap by 46 bp in the (presumably very well annotated) human mitochondrial genome. This suggests that moderate overlaps between adjacent genes may be much more prevalent than currently annotated.

Stop codons located within PCGs are the exception. These are likely cases of annotation or sequencing errors, false assignment of the genetic code, or instances of RNA editing rather than *bona fide* variations of the genetic code itself. However, the number of cases is too small to allow for a conclusive analysis.

A listing of code tables, as provided by the NCBI Taxonomy (22), is indisputably useful. It has a number of shortcomings, however:

1. Some exceptions are only described in the notes of the NCBI code table web page which can not be properly assessed by automated approaches. For example, in the mitochondrial genomes of several arthropods AGG is translated as lysine or arginine instead of serine (26).
2. The additional notes have no claim to be exhaustive. Incomplete stop codons, for example, are only mentioned for vertebrates.
3. Some translational exceptions might be due to taxonomic attributions of the genetic codes that are too coarse-grained.
4. Specifics of the mitochondrial translational system, in particular non-canonical start and incomplete stop codons, are not represented in the codon tables. Representing such properties in the binary genetic code tables might be a non-trivial task.
5. Due to the binary nature of the code tables, the actual prevalence of the usage of start and stop codons is not represented. Rather the mere possibility that a certain codon might be a start is indicated.

Using start and stop codons as defined by the NCBI code tables for annotation, as it was the case in the original implementation of MITOS, is thus not sufficiently accurate. At the same time, our results show that additional efforts should be undertaken to identify the start and stop codons of metazoan mitogenomes. This could be achieved by different strategies:

1. Start and stop codons could be determined by wet-lab experiments modifying the primary sequence. This might be feasible and sufficient for a sample of well chosen key taxa.
2. RNA-Seq or EST data are available for many different species. Previous studies on drosophilids and sauropsids have shown that this data can be used to reliably identify gene boundaries (27,28).
3. Alternatively, the frequencies of annotated start and stop codons can be used for a systematic computational prediction. In this respect the presented study is a first step in this direction.

#### Precise annotation of PCG boundaries

The method presented here identifies start codon positions based on empirical evidence. This also includes non-canonical start codons because they are currently a well-accepted hypothesis (7). While the occurrence of out-of-frame start codons might be plausible, they seem to appear only rarely. Currently, not much is known about the underlying mechanism. Thus, for now, considering them in an automated context would only significantly increase the number of putative start codon positions that need to be checked. Furthermore, the computational prediction of frameshifts close to the 3′ end of PCGs is not a trivial task (20).

In summary, we suggest that translational exceptions (non-canonical start and incomplete stop codons) should be handled with caution, unless accompanied by direct experimental evidence. The possibility of gene overlaps often offers a plausible alternative. The implementation of our new method takes this into account and allows for large overlaps. Similar to any reference-based method, also MITOS might propagate systematic errors that might be present in its reference database RefSeq. In addition to the use of more information than gene similarity MITOS gives additional plots that supports the critical user to evaluate possible alternative start and stop sites, see, e.g. Supplementary Text 1.3 and Figures S4 and S5.

In practice, our method can decide between multiple alternative possible start and/or stop codon positions that seem equally plausible. This is true in particular whenever a single base (i.e. T) is a potential stop codon candidate, which is not an unlikely situation in AT-rich mitogenomes. These cases can be disambiguated by considering the predicted gene length, which can vary significantly between taxa (see Supplementary Figure S6.

Our new method provides consistent and substantial improvements in accuracy over the original implementation in MITOS. The predictions for the test set (additional mitogenomes in RefSeq 89) are of similar accuracy than those for the training set (RefSeq 63, see supplementary material).

Overall, predictions of translation start sites by both, our original and our new approach, differ more with respect to RefSeq than the stop codon predictions. This can be explained by the fact that several start codon positions are possible, whereas – except in cases with incomplete stop codons – the predicted stop codon position is often unambiguous.

Instead of making explicit use of an underlying phylogeny, our method classifies mitogenomes based on their genetic code table. However, it may be desirable to use a more explicit phylogenetic model in future versions of MITOS. Such an extension would make sense in particular if an in-depth analysis shows that mitochondrial PCGs exhibit phylogenetically consistent variations in their start and stop codon positions. For example, it seems plausible that the emergence of overlaps between PCGs and tRNAs is associated with the innovation of stop codons in non-homologous positions. The refined annotations generated by our new method constitute an excellent starting point to address such refinements in a systematic manner.

We note, finally, that the details of PCG annotation of metazoan mitogenomes are mostly based on comparative sequence analysis. As we perform no *de novo* prediction, PCGs that are not annotated in RefSeq, e.g. putative cryptic genes (29), cannot be predicted by our method. Direct evidence from RNA-Seq is rare and available only for a small selection of mitogenomes. Both, the resolution of difficult cases as well as more detailed insights into the evolution of mitochondrial gene boundaries would benefit considerably from additional transcriptomic evidence.

## CONCLUSION

Despite all efforts (30), the RefSeq annotations contain errors (31), in particular implausible assignments of precise start and stop codon positions, and a substantial fraction of incomplete annotations that only identify PCG fragments.

We have demonstrated here that a fully automatic annotation of protein-coding genes in metazoan mitogenomes with very high accuracy is possible, even if employed gene models are inferred automatically from training sets that are not perfect.

The method is implemented online in the MITOS^2^ webservice (http://mitos2.bioinf.uni-leipzig.de) that will be presented elsewhere.

## Supplementary Material

gkz833_Supplemental_File
