# Supplementary material for: Improved annotation of protein-coding genes boundaries in metazoan mitochondrial genomes
Source: Nucleic Acids Res. 2019 Oct 4;47(20):10543–52. doi: 10.1093/nar/gkz833 (PMC6847864; doi:10.1093/nar/gkz833)
Supplement: gkz833_Supplemental_File [file gkz833_supplemental_file.pdf]

Supplementary Material for

Improved Annotation of Protein-Coding Genes Boundaries in Metazoan Mitochondrial Genomes

Alexander Donath, Frank Jühling, Marwa Al-Arab,
Stephan H. Bernhart, Franziska Reinhardt, Peter F. Stadler,
Martin Middendorf, Matthias Bernt*

*Corresponding author: m.bernt@ufz.de

1 Supplementary Text

1.1 Computation of δ and relative start and end positions

In the following, the computation of the factor δ is explained in more detail. Let us first consider the computation of the relative start position $r_s(p)$, which gives the position on the query model that corresponds to position p on the (target) sequence. $r_s(p) = q_s + \frac{q_e - q_s}{s_e - s_s}(p - s_s)$ is computed with respect to a hit (computed with HMMER) with a query model on a sequence. This hit has four coordinates, i.e. the start and end positions in the sequence (s_s and s_e) and the start and end position in the query model (q_s and q_e), see Figure S1.

Consider first the fraction $\frac{q_e - q_s}{s_e - s_s}$. It gives the relation of the length of the query and the length of the sequence that are covered by the hit. This value can be interpreted as how much the hit is stretched or compressed in the sequence with respect to the query (compressed for values > 1 and stretched for values < 1). Next, $p - s_s$ gives the offset between p and the start of the hit in the sequence. The multiplication of $\frac{q_e - q_s}{s_e - s_s}$ and $p - s_s$ translates this offset to query positions, i.e., to the offset between q_s and the sought query position. Hence, adding q_s gives the sought value.

While $r_s(p)$ gives the position on the model that corresponds to p in the sequence, i.e. the offset to the start of the model, $r_e(p)$ gives the offset to the end of the model. Thus, for the computation of $r_e(p)$ $l_q - q_s$ is added instead of q_s.

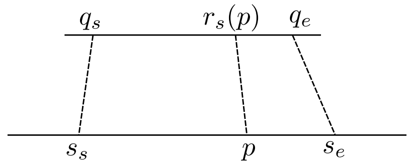

Supplementary Figure S1: Illustration of the positions in the query and sequence used for the computation of $r_s(p)$

For the computation of δ ($\delta_s(p) = 1 - \frac{r_s(p)}{l_q}$ and $\delta_e(p) = 1 - \frac{r_e(p)}{l_q}$), the relative start and end positions, respectively, are normalized with the length of the query model l_q which gives a value between 0 and 1 and measures the distance to the model start and end. One minus this normalised relative positions gives the final δ value.

The rationale behind δ is to penalize positions in the sequence that are covered by the hit that are further away from the query model ends, i.e. further within the query model. δ does not distinguish between position that are not covered by the hit because in these cases δ is set to 1. This is also motivated by the observation by (1) that the hits of the model are mostly shorter than the actual sequence, i.e., positions supported by the hit should belong to the PCG.

1.2 Case study – Bank vole

The mitogenome of the bank vole *Myodes glareolus* (Chordata: Mammalia) (NCBI accessions NC_024538 and KF918859) (2) has been investigated using RNA-Seq data to analyze polyadenylation of the 3' ends of PCGs (3). This allows for a precise annotation of the stop codons. See also Supplemental Text 1.3. Thus, we selected this example to analyze the performance of the novel annotation method for gene boundaries in detail.

Only for three of the 12 PCGs the gene boundaries predicted by the method currently implemented in MITOS are in agreement with RefSeq. Furthermore, for three genes (*nad5*, *nad4*, and *cob*) the annotation of start and stop codon positions by MITOS differs considerably (by more than 18 bp) to the reference annotation. In contrast, the predictions based on our new method are equal to the reference annotation in almost all cases. For three genes stop codon positions differ by only one or two base pairs, because RefSeq includes a full codon in the annotation even if the stop codon is incomplete. The prediction of start codon positions of *nad1* and *nad5* differ by 3 and 9 bp, respectively. Here, the annotation in MitoAnnotator is in agreement with the RefSeq annotation, which thereby avoid overlaps with tRNAs in both genes. But note that overlaps of PCGs with tRNAs are a common feature in mitogenomes according to RefSeq (see Supplementary Figure S11).

To assist the user in the critical evaluation of the proposed annotation, the prediction values of the novel method can be plotted and visually inspected (see Supplementary Figures S4 and S5 for plots for the *nad5* gene of *M. glareolus*). For example, the start codon position of the *nad5* RefSeq annotation (base pair 11 720) differs from the prediction by our new method (base pair 11 711) (see Supplementary Figure S4). However, in both cases, the respective codons are equal (`ATA`). Both codons are located upstream of the initial prediction by HMMER which is

located at position 11 840, which can be seen by δ values of 1, i.e. δ does not help to distinguish these two possible start sites candidates. Furthermore, the plot shows that there are many possible start and stop sites from which the method needs to choose. The predicted stop codon position corresponds to the RefSeq annotation (base pair 13 532) which corresponds to the full stop codon `TAA`, which has a very high empirical probability (ϕ).

The final selection of the start and stop codon position is strongly influenced by the values of λ (see left panel of Supplementary Figure S5). For instance, all combinations that include the putative start position 12 656, which has the largest value for ϕ, have $\lambda = 0$ (which is therefore not shown in Supplementary Figure S5). Despite the small difference of 9 bp, the resulting p-values can differentiate between alternatives ($\lambda = 0.59$ for positions $11711 - 13531$ vs. $\lambda = 0.25$ for positions $11720 - 13531$). The combination of the values for ϕ, δ, and λ leads to a preference for the starting position 11 711 over the RefSeq annotation at position 11 720 (see right panel in Supplementary Figure S5). Thus, the prediction by our new method suggests that *nad5* may overlap with the adjacent *trnL1* gene by 9 bp. Note, that the combinations with the highest values for λ (11 699 with 13 521 ($\lambda = 0.91$) and 13 532 ($\lambda = 0.85$), respectively) are not chosen by our method because the corresponding values of ϕ for the alternative start position are lower (0.03) than for the combination that is finally selected (0.18).

1.3 Protein coding gene boundary prediction from RNASeq data

Gene annotations in reference data bases such as RefSeq or annotations derived from automated or systematic reannotation efforts such as MitoAnn or MitoZoa, respectively, are only an approximation of biological reality. Hence, the explanatory power of a comparison of gene predictions with such data bases might be considered limited. Since the true gene boundaries are unknown for nearly all of the available mitochondrial genomes, these data sources are nevertheless valuable due to the large number of included data. Furthermore, several arguments can be made that the limitations of comparisons with published annotations are not too severe:
1) Annotations in RefSeq have been determined with a multitude of methods and have been ideally scrutinized by different experts on the field. Hence, the chance of systematic errors should be low and non-systematic errors should be compensated by the large number of data sets.
2) Automated analyses in MitoAnn implement state of the art knowledge on gene boundary properties (i.e. present codons).
3) Annotations in MitoZoa are systematically improved reannotations by experts on the field.
4) In the combined analyses with all data sources potential disadvantages of each data source are likely compensated, e.g. inconsistencies in RefSeq annotations due to the usage of a variety of annotation methods in RefSeq annotations and potentialy biased annotations in MitoAnn due to the usage of a consistent annotation method.

In principle, RNASeq data sets offer the possibility to determine real gene boundaries, see below. But a systematic and comprehensive comparison is still difficult:
1) The coverage does not drop sharply at gene boundaries. Instead, a gradual increase or decrease is observed. This is likely because the RNASeq data contains unprocessed and/or only partially processed transcripts. A naïve analysis of the data, e.g. by determining the minimal in the coverage curve, therefore cannot achieve the positional accuracy necessary to improve the annotation of gene boundaries. A method for the accurate inference of gene boundary from coverage data would require a comprehensive understanding of the stochasics of transcript processing. At present, such a quantitative model is not available. It is not at all clear, furthermore, to what extent there are differences between individual species and/or genes, and to what extent the processing is affected by rearrangements of the mitogenome.
2) RNASeq data is only available for a very small fraction of the species in the mitochondrial RefSeq data set.

Hence we focused here on the detection of polyadenylation in RNASeq data which allows to determine 3' gene boundries. In contrast to processing sites, polyA sites can be determined with high accuracy from the available RNAseq data. Our analysis below makes the explicit assumption that the polyadenylation sites mark the transcript end or complete stop codons.

organism	Phylum	ENA ID	genome	RefSeq	# reads	paired	reads on chrM	# polyA
Mus musculus	Chordata	GSM2576079 (GEO)	mm10	NC_005089	23,990,187	n	200,447	11
Castor canadensis	Chordata	ERR2220011	GCF_001984765	NC_033912	30,977,152	y	6,001,520	9
Drosophila melanogaster	Arthropoda	SRR1282418	GCF_000001215.4	NC_024511	144,237,529	n	17,408,891	17
Sitophilus oryzae	Nematoda	SRR2034797	GCA_002938485.1	NC_030765	21,752,880	y	3,113,127	12
Ancylostoma ceylanicum	Nematoda	SRR2230489	GCF_001984765.1	NC_035142	43,193,401	y	3,539,871	8
Octopus bimaculoides	Mollusca	SRR2045866	GCF_001194135.1	NC_029723	27,849,344	y	583,225	17

Supplementary Table S1: Used RNASeq data sets and their properties: organism name, ENA identifier of the RNASeq data, accession of the genome used for mapping, RefSeq accession of the mitochondial genome, total number of reads, paired end (y) or single end data (n), number of reads that mapped to the mitochondrial genome, and number of PolyA sites identified on the mitochondrial genome (above 5% of the coverage)

We downloaded RNASeq data sets from the European nucleotide archive (ENA, `https://www.ebi.ac.uk/ena`) of organisms not in our training set (see Table S1). To obtain a set of phylogenetically diverse organisms, we used data from the insect *Sitophilus oryzae*, the mollusc *Octopus bimaculoides*, the mammal *Castor canadensis*, and the nematode *Ancylostoma ceylanicum*. We also used RNASeq data of two model organisms that were part of our training set: *Drosophila Melanogaster*, and used in-house mouse data from (4). Genomic assemblies were downloaded from NCBI, and mitochondrial sequences were added to the genomes where necessary. The FASTQ files were trimmed using Trim Galore (`http://www.bioinformatics.babraham.ac.uk/projects/trim_galore/`) and cutadapt 2.3 (5) and mapped using segemehl (version 0.3.4 (6)) onto genomes downloaded from NCBI `https://www.ncbi.nlm.nih.gov/genome/`. Coverage on the mitochondrial genomes was computed using bedtools ((7)). PolyA sites were identified using an in house python script that identifies reads with non-genomic poly Ts at the 5' end or poly As at the 3'end. To reduce random artefacts a polyA site was only accepted if it was detected in at least 5% of the reads covering the site. Poly A sites are assigned to the genomic position of the first A (3'end) or last T, respectively. Figures were compiled using the Gviz R-package ((8)). Distances of polyA sites to gene annotations were computed using bedtools closest with the "-d" option separately for forward and reverse strands.

The average coverage of the mitochondrial genome is between $\approx$ 10x (*Mus musculus*) and 100x (*Drosophila melanogaster*). Between eight and 17 polyA sites were detected for each of the mitochondrial genomes. The data shows that the 3' gene boundaries annotated in RefSeq and the 3' gene boundaries predicted by the automatic method presented here have comparable, small distances to polyA sites. Occasional large distances are in most cases explained by missing annotations in RefSeq or MITOS. Besides showing that the automatic annotations of the 3' ends have a good precision this also indicates that a comparison with RefSeq annotations is indeed useful for evaluating the validity of gene boundary predictions.

PolyA sites could not be detected for all genes, see Figure S3. For three to eight PCGs no polyA signal was detected. In particular, no polyA site was detected for the nad4l gene. We emphasize, therefore, that using PolyA signals for detecting 3' ends of genes can not help in all cases.

2 Supplementary Figures

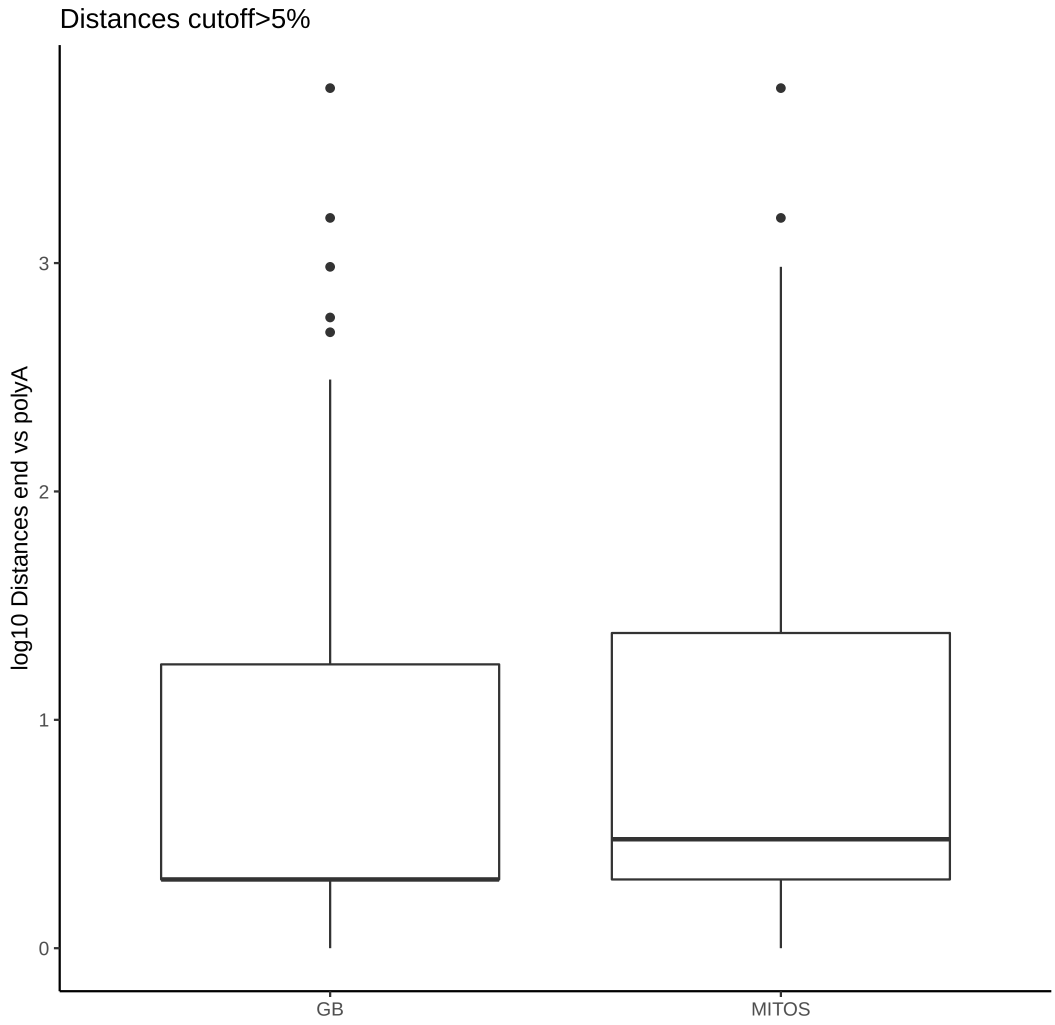

Supplementary Figure S2: Boxplot of the distances of detected polyA sites to the closest 3' gene boundary, for RefSeq (GB) and MITOS.

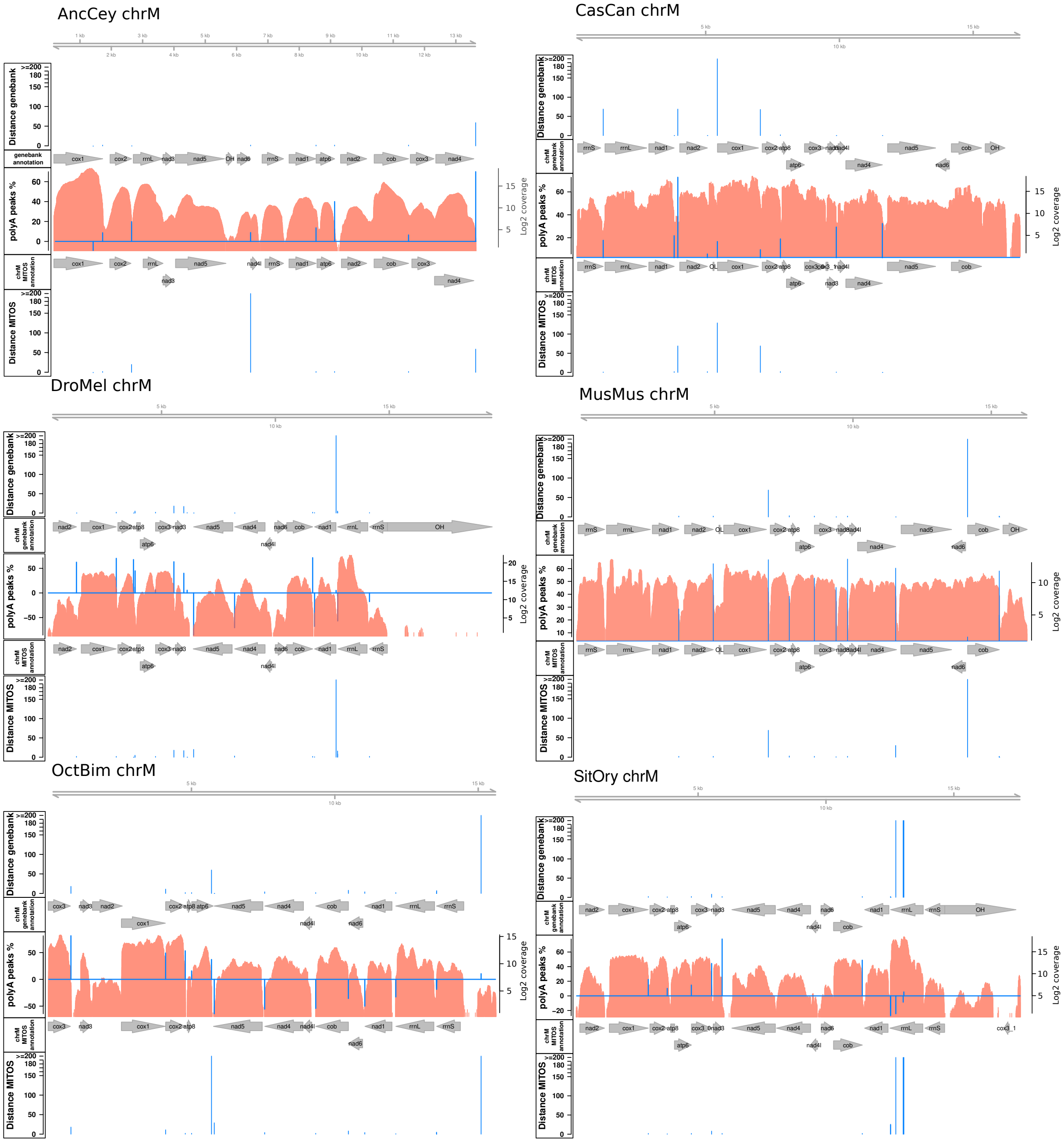

Supplementary Figure S3: Detailed results of the RNASeq analysis for six metazoan species. For each species the plots show the following (from top to bottom) the distance of the polyA sites to the closest 3' boundary annotated in RefSeq (distance genbank), the RefSeq annotations (annotation genbank), the positions and the percent of the observed coverage of the detected polyA sites (polyA peaks %), the MITOS annotations, the distance of the polyA sites to the closest 3' boundary annotated in MITOS (distance MITOS); Species abbreviations are as follows: AncCey: *Ancylostoma ceylanicum*, CasCan: *Castor canadensis*, DroMel: *Drosophila melanogaster*, MusMus: *Mus musculus*, OctBim: *Octopus bimaculoides*, SitOry: *Sitophilus oryzae*.

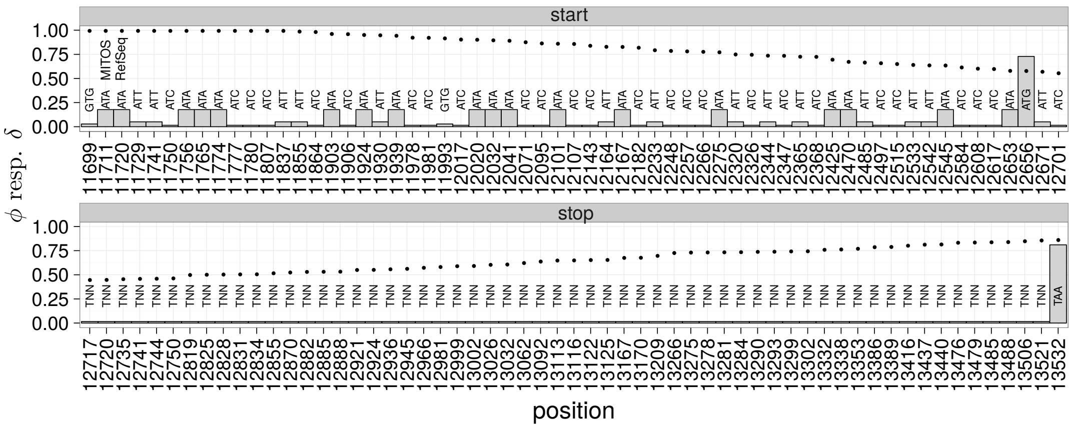

Supplementary Figure S4: Probability values for the codons to be a start or stop codon (ϕ, shown as bars) and the distance of the candidate positions to the estimated start or stop position (δ, shown as points) for the *nad5* gene of the bank vole *Myodes glareolus*. Shown are the values for the gene start (top) and stop (bottom) for positions where both values are > 0. The different start positions chosen by our new method and RefSeq respectively, are indicated. Both methods choose the same base pair position as stop position (13 532).

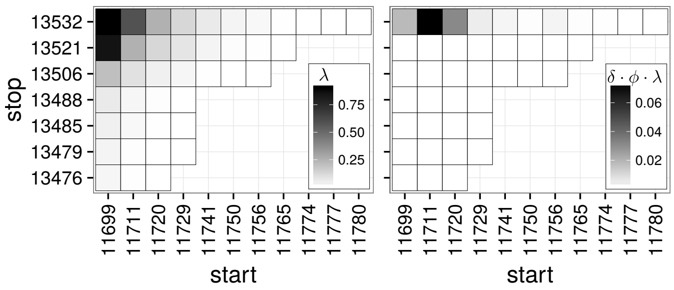

Supplementary Figure S5: Values computed by our new method for the different combinations of potential start and stop sites for *nad5* of the bank vole *Myodes glareolus* (top) p-values of the length distribution for different combinations of start and stop position (bottom) the product of ϕ, δ, and λ from which the maximum is chosen. Only values > 0 are shown in both plots.

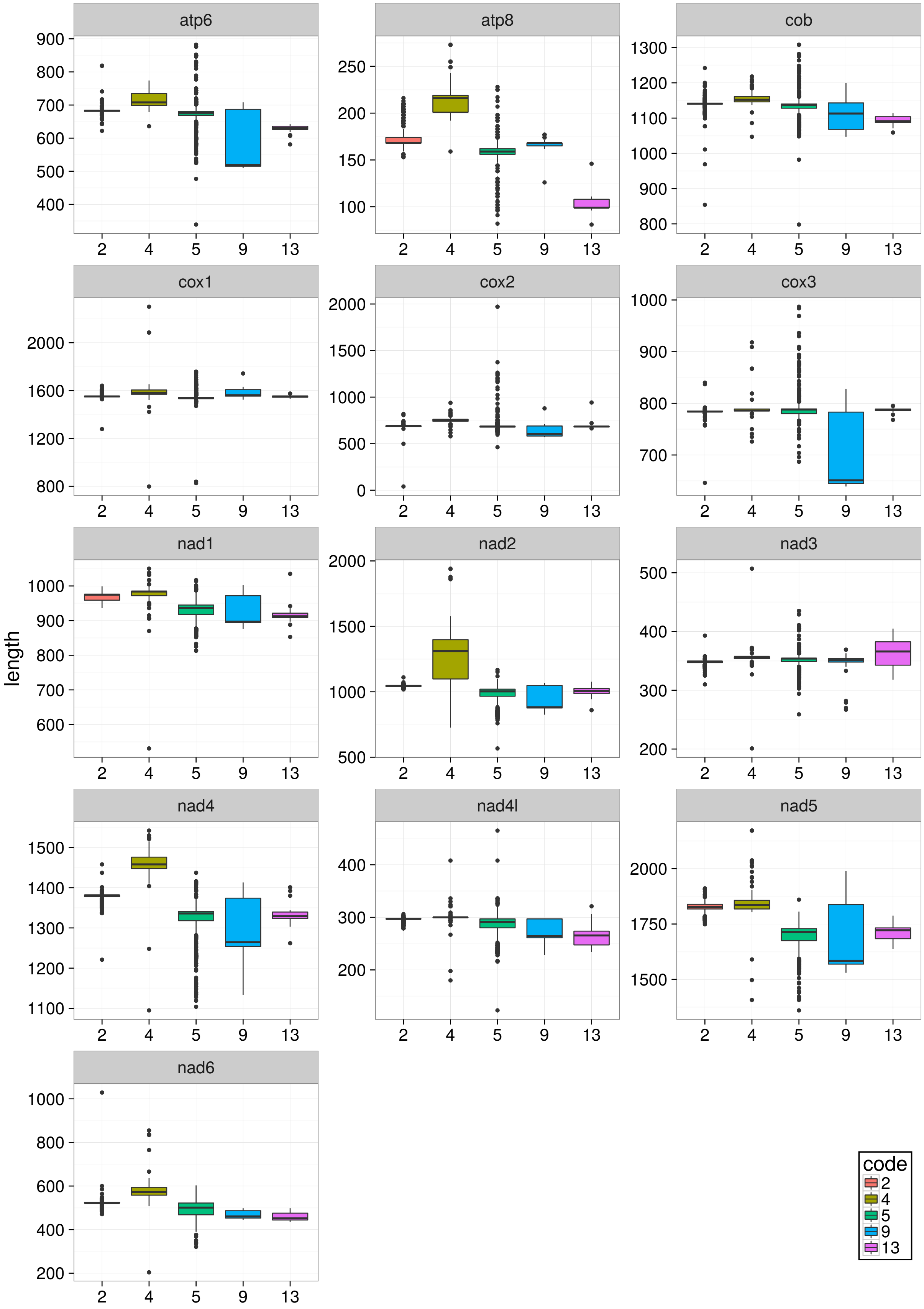

Supplementary Figure S6: Gene lengths (in base pairs) of the mitochondrial protein-coding genes annotated in RefSeq 63.

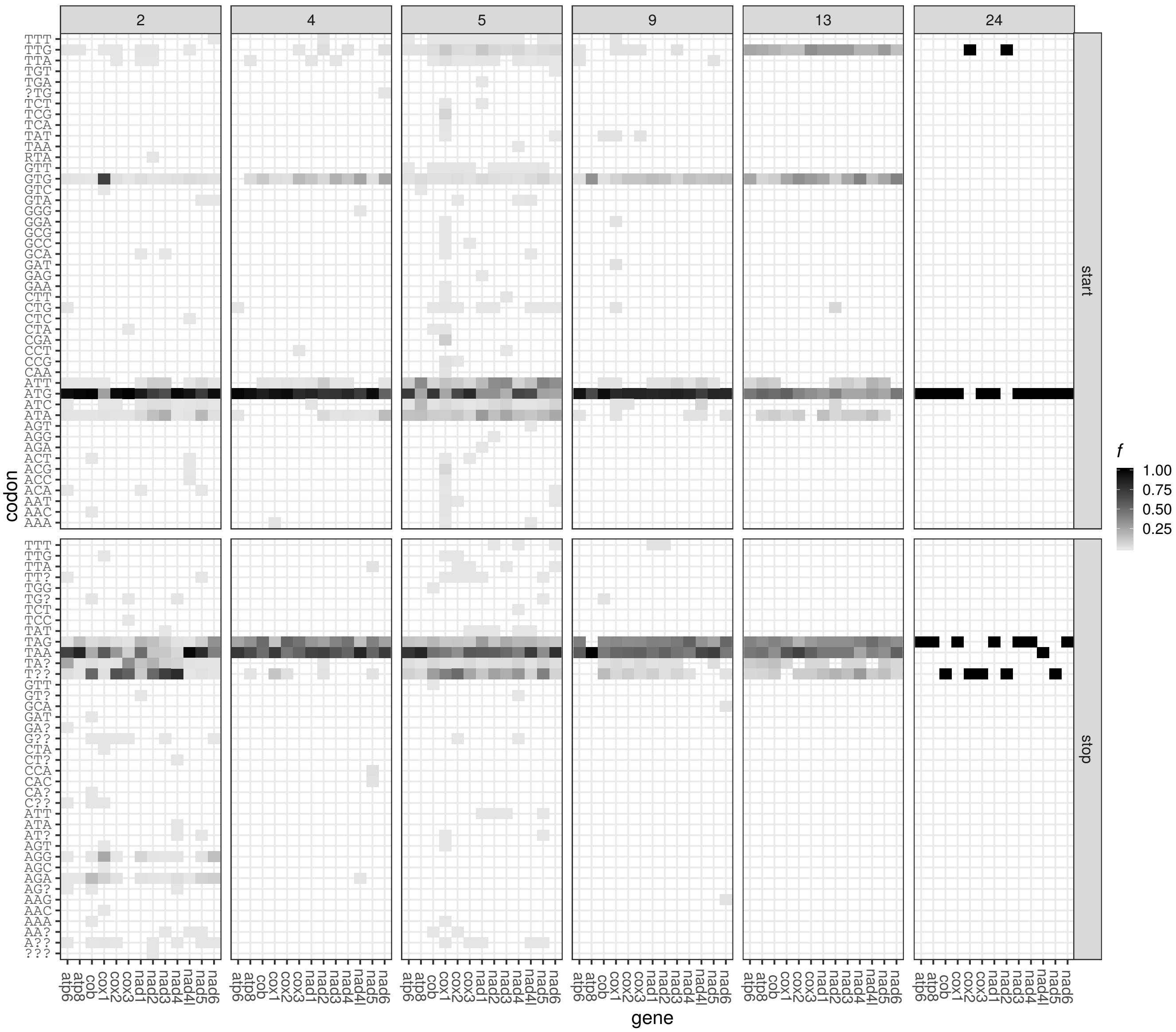

Supplementary Figure S7: Overview of the start codon (upper panel) and stop codon (lower panel) frequencies (f) per gene and genetic code table (2, 4, 5, 9, 13, and 24) inferred from the CDS annotations in RefSeq 63.

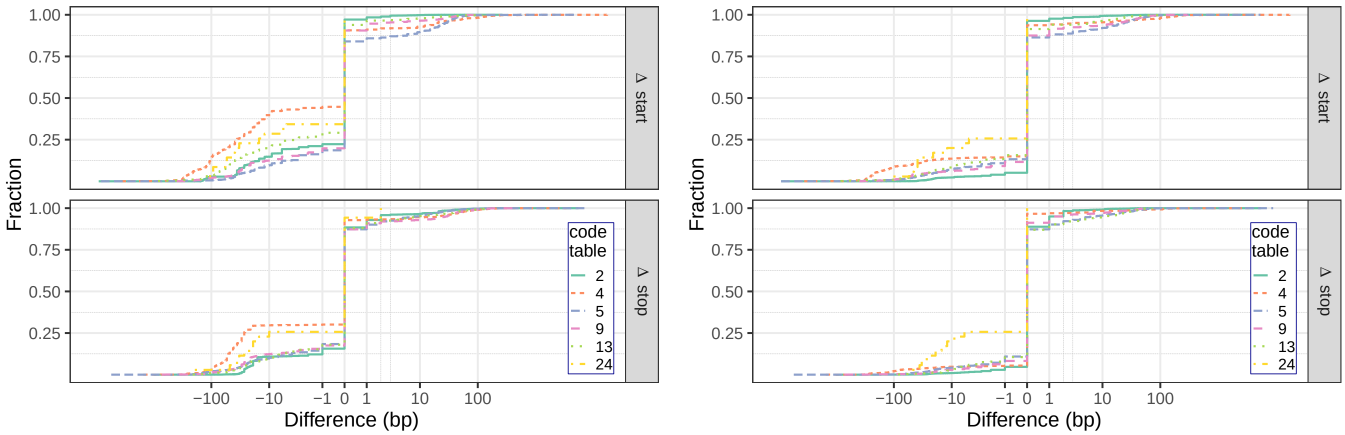

(a) RefSeq 63 (old)

(b) RefSeq 63 (new)

Supplementary Figure S8: Cumulative plot of the differences (in base pairs) of the predicted start and stop codon positions and the positions annotated in RefSeq using the method originally implemented in MITOS (left; "old") and the new method presented here (right; "new") for mitogenomes that are present in RefSeq 63. Positive (negative) values correspond to predictions outside (inside) of the annotation. Differences are shown on an inverse hyperbolic sine scale ($f(x) = \operatorname{arcsinh} x$).

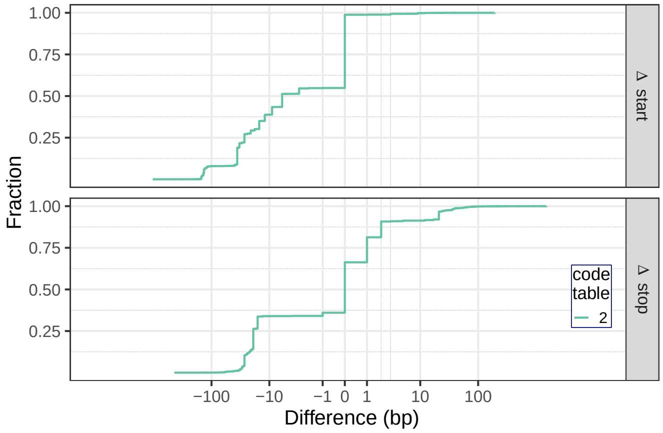

(a) RefSeq 63 (old)

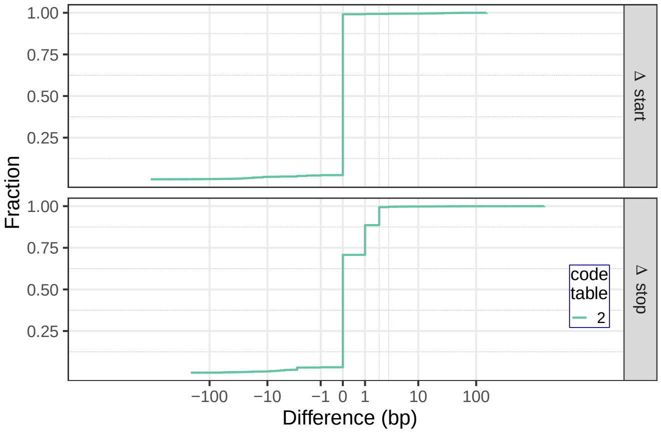

(b) RefSeq 63 (new)

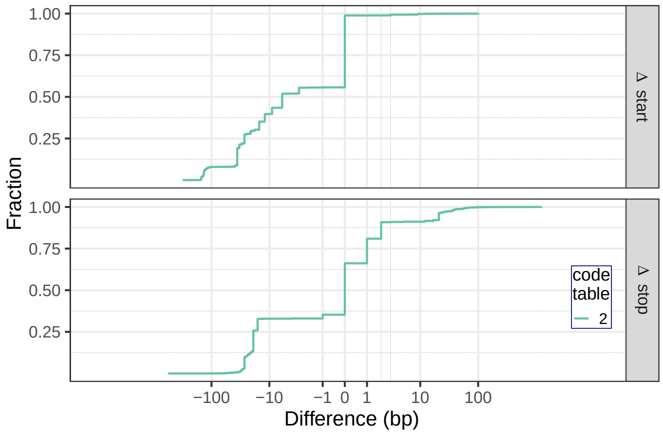

(c) RefSeq 89 (old)

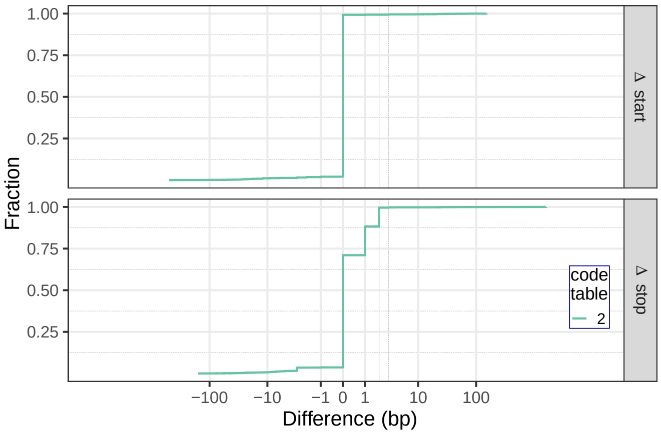

(d) RefSeq 89 (new)

Supplementary Figure S9: Cumulative plot of the differences (in base pairs) of the predicted start and stop codon positions and the positions annotated in MitoAnnotator using the method originally implemented MITOS (left; "old") and the new method presented here (right; "new") for mitogenomes that are present in RefSeq 63 and MitoAnnotator (top) and mitogenomes that are present in RefSeq 89 and MitoAnnotator but not in RefSeq 63 (bottom). Positive (negative) values correspond to predictions outside (inside) of the annotation. Differences are shown on an inverse hyperbolic sine scale ($f(x) = \operatorname{arcsinh} x$).

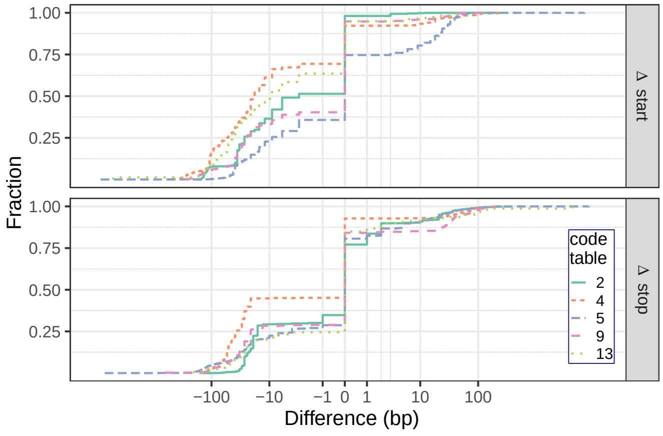

(a) RefSeq 63 (old)

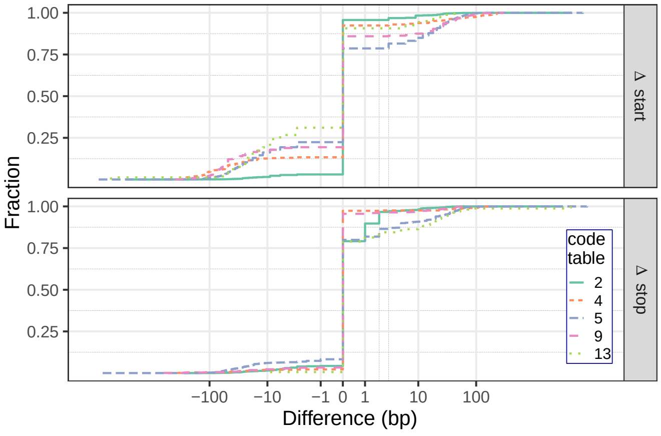

(b) RefSeq 63 (new)

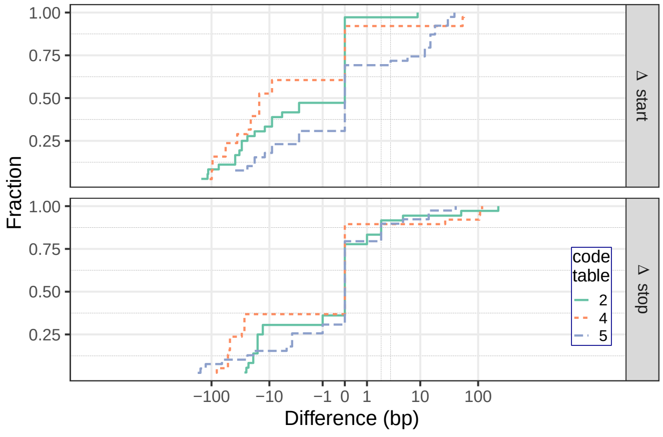

(c) RefSeq 89 (old)

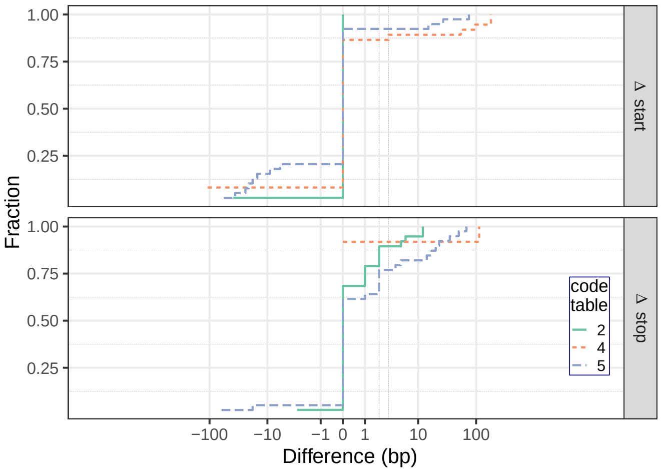

(d) RefSeq 89 (new)

Supplementary Figure S10: Cumulative plot of the differences (in base pairs) of the predicted start and stop codon positions and the positions annotated in MitoZOA using the method originally implemented MITOS (left; “old”) and the new method presented here (right; “new”) for mitogenomes that are present in RefSeq 63 and MitoZOA (top) and mitogenomes that are present in RefSeq 89 and MitoZOA but not in RefSeq 63 (bottom). Positive (negative) values correspond to predictions outside (inside) of the annotation. Differences are shown on an inverse hyperbolic sine scale ($f(x) = \operatorname{arcsinh} x$).

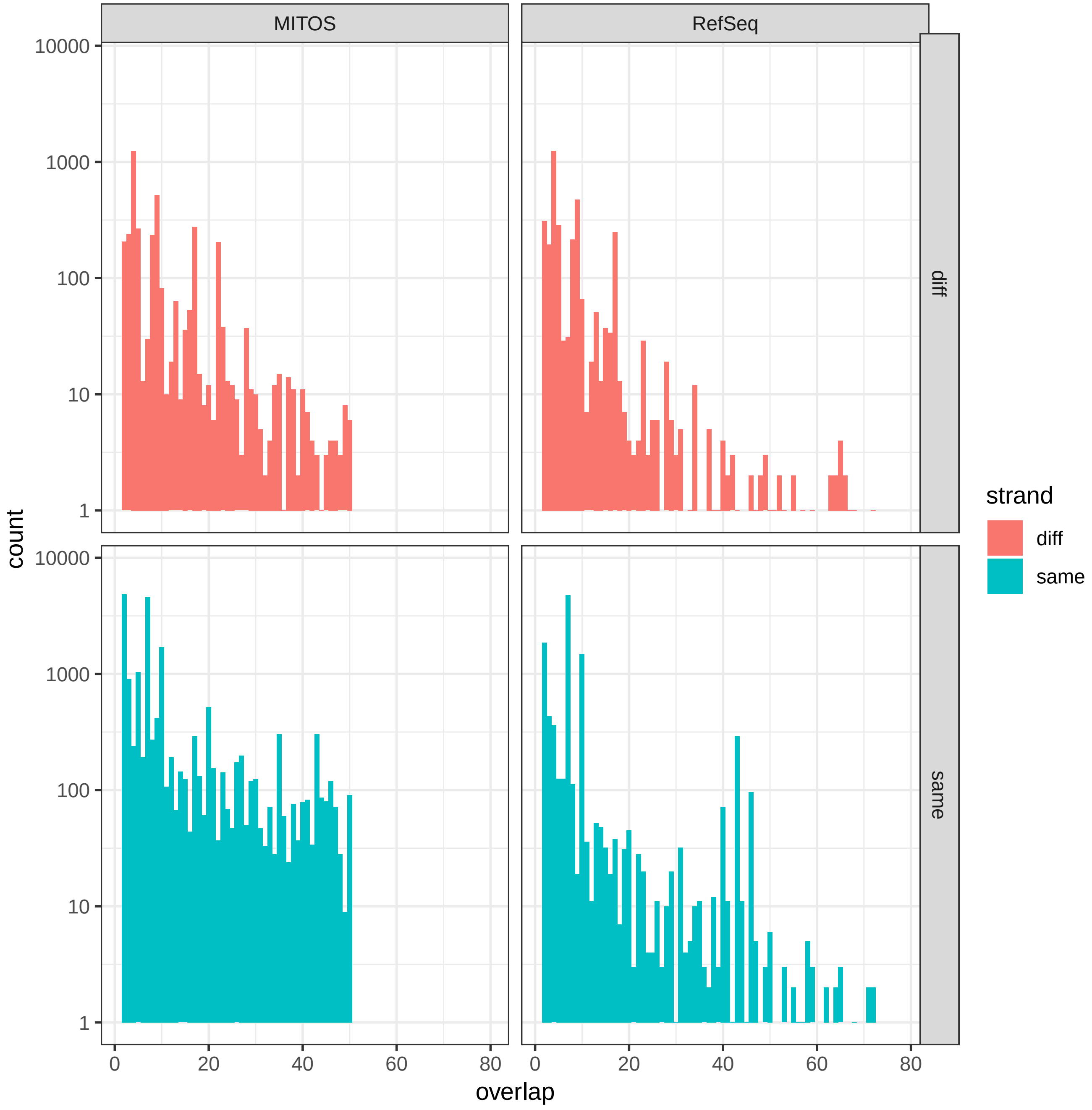

Supplementary Figure S11: Overlaps involving a protein-coding gene in RefSeq 89 (in base pairs) on the same or different (diff) strands. Note that MITOS applies a default maximum overlap of 50bp. In the MITOS annotations 1785 overlaps larger than 30bp out of which 1665 are on the same strand are found. In RefSeq 89 only 689 overlaps of more than 30bp are found out of which 621 are on the same strand.

3 Supplementary Tables

Systematic range	Code table number	Number of mitogenomes RefSeq 63	RefSeq 89
Vertebrates	2	2595	4910
Coelenterates	4	147	211
Invertebrates	5	987	2708
Echinoderm and Flatworm	9	92	169
Ascidia	13	20	26
Flatworm (alternative codetable)	14	0	2
Pterobranchia	24	1	1

Supplementary Table S2: The NCBI genetic code tables used for metazoan mitochondrial genomes and the number of annotated mitogenomes in RefSeq 63 and 89 to which the respective code table is assigned to.

	Equal	Δ±	UP	OP	dif
atp6	4204 (0.96)	1 (0.00)	22 (0.01)	61 (0.01)	71 (0.02)
atp8	4024 (0.83)	4 (0.00)	14 (0.00)	352 (0.07)	480 (0.10)
cob	4251 (0.98)	2 (0.00)	3 (0.00)	43 (0.01)	49 (0.01)
cox1	4261 (0.98)	0 (0.00)	0 (0.00)	57 (0.01)	38 (0.01)
cox2	4253 (0.97)	0 (0.00)	4 (0.00)	65 (0.01)	54 (0.01)
cox3	4256 (0.97)	1 (0.00)	1 (0.00)	45 (0.01)	65 (0.01)
nad1	4235 (0.99)	0 (0.00)	1 (0.00)	16 (0.00)	34 (0.01)
nad2	4218 (0.97)	1 (0.00)	24 (0.01)	37 (0.01)	60 (0.01)
nad3	4246 (0.94)	0 (0.00)	3 (0.00)	200 (0.04)	66 (0.01)
nad4	4252 (0.99)	0 (0.00)	1 (0.00)	22 (0.01)	31 (0.01)
nad4l	4206 (0.97)	0 (0.00)	15 (0.00)	63 (0.01)	52 (0.01)
nad5	4259 (0.98)	0 (0.00)	0 (0.00)	24 (0.01)	67 (0.02)
nad6	3785 (0.95)	2 (0.00)	92 (0.02)	45 (0.01)	53 (0.01)
Σ	54450 (0.96)	11 (0.00)	180 (0.00)	1030 (0.02)	1120 (0.02)

Supplementary Table S3: Quality of annotations using the new method for predicting start and stop codons for PCGs in metazoan mitogenomes contained in RefSeq 89. Shown are the comparisons with annotations provided by RefSeq. The quality of the predictions is determined by identifying for each predicted protein-coding gene (PCG) the corresponding RefSeq PCG that has the bidirectional maximum overlap. Predicted PCGs of that having a counterpart in RefSeq are classified as equal (Equal), if they have the same name (i.e. annotate the same PCG) and are located on the same strand; Δ±, if they have the same name but are located on the opposite strand; or different (dif), if they have a different name (and possibly a strand difference). The remaining predictions that have no counterpart in RefSeq are classified as overpredicted (OP). Finally, features of RefSeq that have no counterpart in our predictions are counted as underpredicted (UP).

	RefSeq 63 (old)		RefSeq 63 (new)	
	start	stop	start	stop
μ	9.15	6.11	4.4	2.26
σ	83.51	56.96	86.98	57.57
$d = 0$	59.20%		75.62%	
$d \leq 3$	70.70%		88.66%	
$d \leq 9$	75.78%		91.68%	
$d \leq 30$	89.14%		96.52%	

Supplementary Table S4: Statistics of the precision of the gene boundary predictions for the method originally implemented in MITOS (old) and the new method presented here (new) for the reannotation of the RefSeq 63 mitogenomes. Top part: mean (μ) and standard deviation (σ) of the absolute values of the differences for start and stop position (in base pairs (bp)). Bottom part: percentage of the genes where the maximum difference (d) between annotated and predicted start and stop positions is less than or equal to 0, 3, 9, and 30 bp, respectively.

	MitoAnnotator (1349)		MitoZOA (2471)	
	start	stop	start	stop
μ	20.26	11.08	19.31	14.27
σ	38.25	25.85	78.88	77.66
$d = 0$	10.12%		18.77%	
$d \leq 3$	28.77%		31.31%	
$d \leq 9$	39.42%		42.03%	
$d \leq 30$	75.31%		73.46%	

(a) RefSeq 63 (old)

	MitoAnnotator (1349)		MitoZOA (2471)	
	start	stop	start	stop
μ	0.92	1.26	6.54	3.74
σ	14.25	21.89	92.14	77.09
$d = 0$	65.99%		63.40%	
$d \leq 3$	96.21%		80.04%	
$d \leq 9$	97.57%		85.20%	
$d \leq 30$	99.17%		93.80%	

(b) RefSeq 63 (new)

	MitoAnnotator (1267)		MitoZOA (9)	
	start	stop	start	stop
μ	20.16	10.58	20.71	19.18
σ	36.47	20.73	31.10	38.30
$d = 0$	10.52%		19.47%	
$d \leq 3$	28.41%		29.20%	
$d \leq 9$	39.67%		40.71%	
$d \leq 30$	76.12%		68.14%	

(c) RefSeq 89 (old)

	MitoAnnotator (1267)		MitoZOA (9)	
	start	stop	start	stop
μ	0.67	1.19	10.34	6.28
σ	9.03	19.91	31.77	20.65
$d = 0$	66.26%		61.4%	
$d \leq 3$	96.91%		74.56%	
$d \leq 9$	98.02%		78.95%	
$d \leq 30$	99.30%		89.47%	

(d) RefSeq 89 (new)

Supplementary Table S5: Statistics of the precision of the gene boundary predictions for the method originally implemented in MITOS (left) and the new method presented here (right) for the RefSeq 63 data (top) and the RefSeq 89 beyond RefSeq 63 (bottom) with respect to the reference annotations in MitoAnnotator and MitoZOA. Top part: mean (μ) and standard deviation (σ) of the absolute values of the differences for start and stop position (in base pairs (bp)). Bottom part: percentage of the genes where the maximum difference (d) between annotated and predicted start and stop positions is less than or equal to 0, 3, 9, and 30 bp, respectively. Numbers in parentheses give the number of mitogenomes used for the statistics, i.e. the number of mitogenomes that appear in the test data and reference annotation and have at least one true positive gene prediction.

References

1. Al Arab, M., zu Siederdissen, C. H., Tout, K., Sahyoun, A. H., Stadler, P. F., and Bernt, M. (2017) *Molecular Phylogenetics and Evolution* **106**, 209–216.

2. Bendová, K., Marková, S., Searle, J. B., and Kotlík, P. (2016) *Mitochondrial DNA A* **27**, 111–112.

3. Marková, S., Filipi, K., Searle, J. B., and Kotlík, P. (2015) *BMC Genomics* **16(1)**, 870.

4. Mages, C. F., Wintsche, A., Bernhart, S. H., and Müller, G. A. (2017) *eLife* **6**, e26876.

5. Martin, M. (2011) *EMBnet.journal* **17(1)**, 10–12.

6. Hoffmann, S., Otto, C., Doose, G., Tanzer, A., Langenberger, D., Christ, S., Kunz, M., Holdt, L. M., Teupser, D., Hackermüller, J., and Stadler, P. F. (2014) *Genome Biology* **15(2)**, R34.

7. Quinlan, A. R. and Hall, I. M. (2010) *Bioinformatics* **26(6)**, 841–842.

8. Hahne, F. and Ivanek, R. (2016) Visualizing genomic data using Gviz and Bioconductor In Ewy Mathé and Sean Davis, (ed.), Statistical Genomics: Methods and Protocols, pp. 335–351 Springer.
